# Breaking down biofilms across critical priority fungal pathogens: proteomics and computational innovation for mechanistic insights and new target discovery

**DOI:** 10.1128/mbio.02303-24

**Published:** 2025-07-22

**Authors:** Oscar Romero, Jennifer Geddes-McAlister

**Affiliations:** 1 Department of Molecular and Cellular Biology, University of Guelph, Guelph, Ontario, Canada; Instituto Carlos Chagas, Curitiba, Brazil

**Keywords:** biofilms, critical priority fungal pathogens, mass spectrometry-based proteomics, computational methods, drug discovery

## Abstract

Fungal biofilms are complex microbial structures associated with persistent and progressive infections, such as cryptococcal meningitis, invasive aspergillosis, and invasive candidiasis, leading to thousands of deaths annually. The prevalence of fungal biofilm formation during infections, with its heightened resistance to antifungal drugs, highlights the urgency for the discovery and development of new antifungals with antibiofilm activity. Current advances in mass spectrometry-based proteomics and computational platforms provide a powerful toolkit to accelerate drug discovery from target identification to optimization of a lead molecule. In this review, we highlight fungal biofilms of four critical priority fungal pathogens (as deemed by the World Health Organization) and define important technological considerations for proteomics and computational methodologies. Additionally, we explore recent proteomics and computational applications within fungal biofilms for the identification and elucidation of biological mechanisms underscoring biofilm formation as well as the discovery of novel putative antibiofilm targets.

## INTRODUCTION

Fungal diseases are a growing threat to global health, with over 6.5 million infections reported annually, leading to approximately 3.8 million deaths (1). Fungal-related deaths surpass those caused by tuberculosis and malaria combined (2, 3), and yet, fungal diseases are vastly underreported and misdiagnosed, leading to challenges in treatment and priority (4). In addition, the prevalence and severity of fungal infections are increasing with new emerging pathogens, their distribution across new geographical regions and host niches, and rising rates of antifungal resistance through intrinsic and evolutionary mechanisms (5, 6). Hence, in 2022, the World Health Organization (WHO) released the inaugural Fungal Priority Pathogens List, which classifies fungal pathogens in different priority levels depending on antifungal resistance, mortality rates, evidence-based treatment, access to diagnostics, annual incidence, and complications of previous infections (7). The designated Critical Priority Group consists of *Cryptococcus neoformans*, *Aspergillus fumigatus*, *Candida albicans*, and *Candida auris*.

For these pathogens, infections are most prevalent in immunocompromised individuals; however, infections in immunocompetent individuals due to immune system disruption are on the rise. For instance, *C. neoformans* causes the deaths of over 115,000 people each year through the development of cryptococcal meningitis, whereas *A. fumigatus* causes invasive aspergillosis (IA), leading to over 1.8 million deaths each year, with the severity of these diseases confounded by chronic obstructive pulmonary disease, intensive care unit stays, lung cancer, asthma, and hematological malignancies (1, 8). Conversely, *C. albicans* is a human commensal fungus, part of the human microbiome; however, upon perturbation of the host microbiome and/or immune system, *C. albicans* bloodstream infection or invasive candidiasis can develop, causing approximately 1 million deaths annually (1). *C. auris*, a recently emerged pathogen with intrinsic resistance to multiple antifungal classes, is rapidly contributing to the death rates associated with *Candida* spp. (9).

### Human fungal pathogens on the Critical Priority List

*Cryptococcus neoformans* is a dimorphic fungus of the Basidiomycota phylum; although filamentous morphology may be present, it is typically found during sexual reproduction, which happens in a natural environment (10, 11). Hence, the yeast morphology is more common during infection. The natural niches of *C. neoformans* are bird droppings and decaying wood, where the fungus may be found either as desiccated yeast cells or basidiospores (12), along with reports of fungal survival in aquatic environments (13). The fungal cell morphologies are easily aerosolized, facilitating inhalation and transportation through the host’s upper respiratory tract toward the alveolar sacs, where the infective cells may reside (14). Importantly, in immunocompetent individuals, actions of the innate immune system (e.g., macrophage phagocytosis) and crosstalk with the adaptive immune system (e.g., T cells) permit the clearance of fungal cells from within the lungs (15, 16). However, in immunocompromised hosts, cryptococcal cells may overcome the innate immune response initiated within the lungs and reside undetected within alveolar macrophages, limiting adaptive immune system activation (17, 18). In this sense, *C. neoformans* may survive and proliferate without eliciting further immune responses, and through neurotropism, the fungal cells may disseminate throughout the host to cross the blood-brain barrier and infect the central nervous system to cause cryptococcal meningitis (19–21).

The filamentous fungus *A. fumigatus* belongs to the Ascomycota phylum and is an opportunistic human pathogen. It is typically a cosmopolitan species found in soil from outdoor and indoor environments. Predominantly saprophytic, *A. fumigatus* grows in decaying or dead matter, and like other filamentous fungi, the fungus grows as hyphae and produces haploid conidia by asexual reproduction in conidiophores (22–24). The conidia are small (2–3 µm) and highly hydrophobic, making them readily aerosolized, and upon inhalation, the conidia cross the upper respiratory tract and settle into alveolar spaces (25). Like other fungal pathogens, a healthy immune system can eliminate the infective cells by using alveolar macrophages to phagocytose and destroy the pathogenic cells (26, 27). However, in immunocompromised individuals, *A. fumigatus* conidia may germinate and develop extensive lung mycelium structures during periods of immune suppression (e.g., chemotherapy), leading to the spread and development of IA (28).

Similar to *C. neoformans, C. albicans* is a dimorphic fungus, part of the Ascomycota phyla, and is commonly found as a commensal yeast within the human digestive system and vaginal tract, as part of the microbiota (29, 30). However, *C. albicans* is an opportunistic pathogen that, during periods of immune suppression (e.g., HIV/AIDS) or disruption of the microbiome (e.g., antibiotic treatments), can surpass the innate immune system and infect the habitual niches, causing oral and vaginal thrush (31, 32). In severe cases, the yeast may spread throughout the body, causing acute disseminated *Candida* septicemia and invasive candidiasis (33–35). *C. albicans* exists in diverse morphologies, including the blastospore found during commensality, compared to pseudohyphae (i.e., chains of elongated yeast cells) and true hyphae (i.e., branched filaments of tubular cells) found during infection (36, 37). Fungal cell wall compositions differ among the morphologies, influencing virulence and evasion of the host immune system (38, 39).

*Candida auris* was first described in Japan (2009) and shares a close phylogenetic relationship with the emerging, multidrug-resistant species *Candida ruelliae* and *Candida haemulonii* (40). Since its discovery, multiple isolated cases of *C. auris* infection have been documented worldwide, and separate clades have emerged simultaneously (41–44). The emergence of this non-*albicans* species has been attributed to climate change due to the anthropogenic pressure on the environment, which may cause improved thermotolerance of the fungus, supporting growth at higher temperatures (40°C) (45, 46). Notably, *C. auris* is characterized by nosocomial infections and has intrinsic resistance to commonly used antifungal agents used to treat candidiasis, including azoles, polyenes, and echinocandins (47–50). Contrary to *C. albicans,* the pseudohyphae morphology in *C. auris* has only been reported upon induced DNA damage (51).

### Antifungal therapies and resistance—the importance of new drugs

Current treatment of fungal diseases relies on four main classes of antifungal drugs: azoles, polyenes, echinocandins, and pyrimidine analogs (52, 53). Depending on the fungal species, some classes are more effective than others (54). For example, azoles are the most widely used antifungal drugs in clinical and agricultural settings due to the different azole drugs available, initial effectiveness, availability, and low cost (55–57). The mechanism of action for azoles promotes the inhibition of lanosterol 14-α-demethylase, an enzyme involved in synthesizing ergosterol, a key component of the fungal plasma membrane (58, 59). Thus, inhibition of this enzyme leads to the accumulation of toxic ergosterol intermediates and plasma membrane impairment. However, given the fungistatic (i.e., growth inhibition) nature of azoles, which exerts a high selection pressure on the fungal cell, the development of resistance is prominent (60). In agriculture, azoles control fungal diseases of crops and act as a prophylactic, driving the development of new strains with resistance to azoles (57, 61). Moreover, the prolonged or inappropriate use of antifungals may give rise to new pathogenic species with intrinsic resistance, such as *C. auris*, with a resistance rate of 86% toward azoles (9, 62). Furthermore, the emergence of *C. neoformans* fluconazole-resistant strains has increased in resource-limited countries, where the drug is the sole treatment option for cryptococcal meningitis (63–65).

Another drug of choice to treat mycoses is amphotericin B (a polyene), which interacts with ergosterol localized in the fungal cell membrane, producing a pore complex. The formation of pores across the membrane causes leakage of cellular compartments and, consequently, cell death (66). However, this fungicide may also target the cholesterol found in host cells to (67) exert a high nephrotoxicity, even at low concentrations, requiring hospitalization for proper drug administration (68, 69). Resistance to amphotericin B is less common, although still present, with *C. auris* demonstrating a resistance rate of 26% to amphotericin B (70, 71). Another antifungal agent with fungistatic activity is the pyrimidine analog, 5-fluorocytosine (5FC), which interferes with DNA and RNA synthesis (72). However, monotherapy with 5FC is discouraged as the pathogen rapidly develops resistance (73). Instead, 5FC is used in combination with other antifungal drugs, such as amphotericin B, as initial therapy for cryptococcal meningitis (74, 75). It may also be used for invasive candidiasis as part of a combination therapy (76, 77). Finally, echinocandins target the 1,3-β-D-glucan synthase, a key enzyme for β-glucan synthesis, a central component of the fungal cell wall (78). Without β-glucans, the fungal cell wall is destabilized, leading to cell death. This antifungal class displays both fungistatic and fungicidal properties depending on the pathogen and is mainly used to treat candidiasis, as *Candida* species are more susceptible, though echinocandins may also be used in combination with azoles and polyenes to treat IA (79, 80). Notably, echinocandins are ineffective against *Cryptococcus* spp. since these fungi are intrinsically resistant to the antifungal class (81, 82). Overall, the emergence of new fungal species and strains resistant to available antifungal drugs underscores the urgent need for developing new therapies.

### Classical fungal virulence factors

Fungal pathogens rely on various virulence factors to initiate and maintain infection, as well as resist host defenses (83). Virulence factors contribute to various processes essential for infection, including colonization, growth at host body temperature (37°C in humans), nutrient acquisition, immune evasion, and host tissue damage. The presence and efficacy of these factors determine the pathogen’s level of virulence. For example, pathogens possess thermotolerance to survive within the host, as observed in *C. neoformans*, *C. auris*, and A. *fumigatus*, which are environmental fungi capable of adapting and growing at elevated temperatures (i.e., 37°C) (84–86). Another example includes morphologic switching, as observed in *C. albicans*, to trigger the release of virulence factors (e.g., candidalysin, a hyphae-specific toxin) to promote infection (87, 88). The distinct morphologies may also facilitate cell wall remodeling, a critical aspect of host-pathogen interactions (89). This process enhances direct contact with the external environment, allowing fungal surface proteins (e.g., glycoproteins) to interact more effectively with proteins on the host tissue (90, 91). Notably, for *C. neoformans*, its cell wall surface is not in direct contact with the surroundings due to the presence of a prominent polysaccharide capsule, which interacts directly with the external environment (92). The capsule is a main virulence factor of *C. neoformans*, conferring protection against the host’s immune system and enabling the colonization (93). Another virulence factor critical for colonization is the secretion of adhesins, which recognize surface proteins on host tissue for effective binding and attachment to promote invasion (94). The secretion of exoenzymes, such as proteases, lipases, and nucleases, is also a key factor in obtaining nutrients and damaging the host tissue (95–98). *C. neoformans*, *A. fumigatus*, *C. albicans*, and *C. auris* use such enzymes to disseminate throughout the body and colonize different tissue types and evade the host immune response (99). Among other virulence factors used by pathogenic fungi are the production of toxins (e.g., gliotoxin by *A. fumigatus*), melanin production, and stress response proteins (e.g., superoxide dismutase) (100, 101).

### Fungal biofilms with diverse roles in pathogenesis and virulence

**Fig 1:**
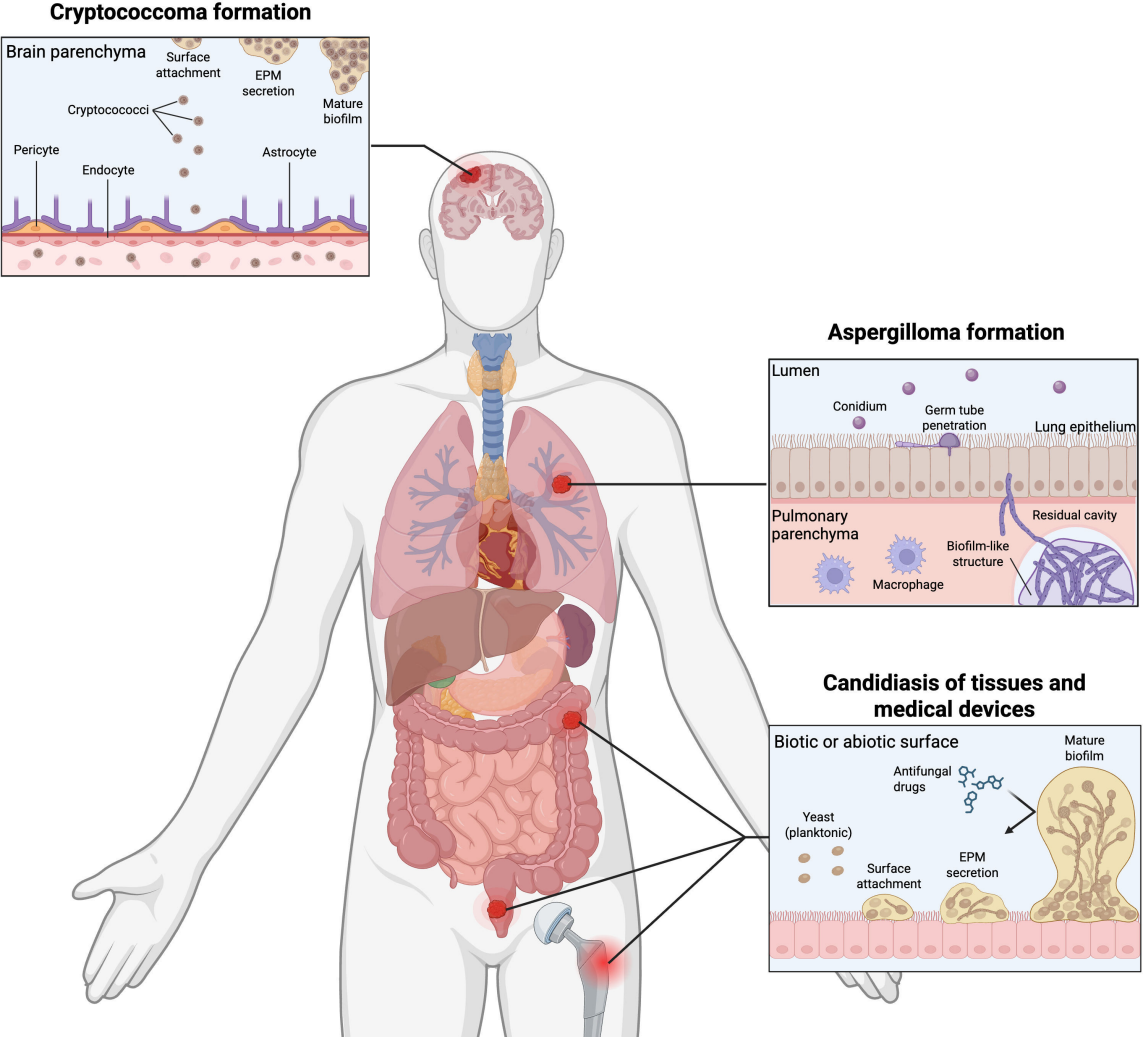
Biofilm formation as a mechanism of infection by *C. neoformans*, *A. fumigatus*, and *C. albicans*. Cryptococcoma formation is initiated by cryptococci crossing the blood-brain barrier through different mechanisms (i.e., paracellular transmigration). Once in the brain, the yeast cells attach to a surface, forming a basal layer, followed by the extracellular polysaccharide matrix secretion. For *A. fumigatus*, after inhalation, the conidia may lodge in pre-existing cavities caused by previous diseases in lung tissue, where they germinate, and after hyphae formation, an EPM is produced, gluing the filaments together and producing a fungal ball (aspergilloma). *C. albicans* can colonize biotic and abiotic surfaces through biofilm formation, with yeast cells forming an initial basal layer to undergo a morphological switch to hyphae for the production of EPM.

In harsh environments, microorganisms often organize into biofilms, which are aggregations of cells embedded in an extracellular polysaccharide matrix (EPM), rather than individual cells to promote survival (102, 103). The EPM determines the consistency and properties of the structure, since composition varies depending on the microorganism (104). For example, *C. neoformans* biofilm is mainly composed of glucuronoxylomannan, a central component of the capsule (105, 106). Pathogens may develop biofilms during active infections to facilitate colonization, increasing disease severity, or during latent periods of the infection to persist within the host (Fig. 1) (107–109). Within biofilms, fungal cells have greater protection from detection and destruction by the host immune system and against antifungal therapies, which drives the need for increased drug concentrations, supporting cytotoxicity towards the host (110, 111). Therefore, biofilm colonization increases the morbidity and mortality of fungal infections (112).

In *Candida* spp., biofilm formation is common during infections for improved attachment to host tissue surfaces throughout the body (113). *Candida* biofilms are especially complex due to a morphological switch to hyphae, leading to multiple morphologies within a single biofilm that increase resistance to immune system defenses (114, 115). Biofilm formation begins with the attachment of *Candida* spp. cells to either biotic or abiotic surfaces, making the basal layer of the biofilm (116, 117). Then, cells proliferate and start the filamentation until hyphae are formed, followed by the production of EPM that surrounds the cells and builds an internal environment (118). Biofilm maturation occurs with EPM accumulation and increased cellular growth, followed by detachment of yeast cells from the biofilm to promote dispersal to new locations for the formation of additional biofilms (119). Similarly, *C. neoformans* promotes biofilm development using the same strategies but maintains a single yeast-like morphology (120). Cryptococcal biofilm formation is a hallmark of brain tissue colonization during cryptococcal meningitis, known as cryptococcomas, and is sometimes misdiagnosed as brain tumors (121–123). Moreover, in the clinical settings, cryptococcal biofilms that form on medical devices (e.g., shunts) promote fungal survival and impair disease management strategies (124). Conversely, *A. fumigatus* biofilms begin with a basal layer formed by conidia deposited on the alveolar surface, which germinate into hyphae (125, 126). Once the hyphae are attached, they secrete a strong EPM until maturation, which works like a glue to form a tight hyphal network and complex structure (127). Overall, the formation of fungal biofilms is a critical determinant of disease severity and outcome, with many cellular and molecular processes regulated by the production of proteins that drive morphological changes, metabolic shifts, biofilm organization, and EPM secretion. Understanding the role of these proteins and characterizing their potential as novel antifungal targets are crucial in advancing our knowledge and ability to overcome fungal biofilms.

## INNOVATIONS IN THE DRUG DISCOVERY PIPELINE

Drug discovery is a collaborative endeavor primarily rooted in chemistry and supported by pharmacology and biological and clinical sciences (128, 129). The process of drug discovery is guided by three elements (i.e., drug, target, and disease phenotype) toward the development of bioactive molecules that interact with specific biological targets. Such interactions aim to change disease phenotypes. Depending on the strategy, the drug discovery process may begin with two initial points: identifying a bioactive molecule or selecting a biological target. Historically, most first-in-class drugs were isolated from medicinal plants and used empirically for treatment without knowledge of their targets (130, 131). Later, the development of new technologies and collaboration across disciplines, such as biochemistry and structural biology, enabled the identification of drug targets and elucidation of their mechanisms of action (130).

Modern drug discovery mainly uses target-based strategies as an efficient approach, which has been substantially accelerated by technological advancements (e.g., omics) and the availability of large compound libraries (131–133). However, the fact that a biomolecule is identified as a potential target in a particular disease does not guarantee its suitability for drug intervention (134). Hence, once a biomolecule is linked to a disease, it must be rigorously validated in relevant models, such as animal studies (e.g., mouse), to confirm that activity regulation leads to reproducible phenotypic changes. Once validated, the targeted molecule can be used for high-throughput screening (HTS) of large compound libraries or rational drug design (135). HTS enables the rapid identification of compounds interacting with the target, whereas rational drug design involves creating a molecule based on knowledge of the biological target, which may result in better specificity toward the target. The compounds selected from these processes undergo further investigation through *in vitro* testing to assess bioactivity, and molecules without the desired effects are discarded, narrowing down the initial pool of candidates (136). Finally, optimization of physicochemical and pharmacological properties is conducted once promising hits are identified to enhance efficacy, bioavailability, and safety. The prediction of pharmacokinetic properties may also be important for preclinical information since it provides information about absorption, distribution, metabolism, excretion, and the potential for toxicity (ADMET) in the model of study (137, 138). Excitingly, the drug discovery pipeline is continuously updating with the emergence of novel technologies, such as structure prediction tools using artificial intelligence (AI) and integrative omics (139). These new tools, combined with multidisciplinary collaboration, are accelerating the discovery of necessary drugs, such as antifungals, and paving the way for a more efficient and precise development of therapeutics for current and future medical needs.

### Mass spectrometry-based proteomics

Proteins perform diverse processes within biological systems, such as structural support, communication, regulation, defense, transportation, movement, storage, and catalysis (140). In this sense, proteins also play a critical role in the pathological mechanisms of disease, which makes them suitable druggable targets. Such is the case of virulence factors in fungal pathogens, which may either be synthesized by a protein or the virulence factor is a protein itself (i.e., proteases), making them important targets for drug development. Mass spectrometry-based proteomics provides an avenue to investigate proteins from diverse biological systems on a small or large scale. Applications include protein identification and quantification, functionality, structure, and post-translational modification annotation, which can be applied for different purposes, such as drug discovery (141). In this sense, mass spectrometry-based proteomics plays a crucial role in identifying biological targets, thereby enhancing a target-based approach to drug discovery. In the context of fungal diseases, understanding the mechanisms of pathogenesis through the identification of the proteins involved is a key area of research for developing new therapeutic drugs (98, 142–146). The total proteome of the fungal cell, secreted proteins, and biofilm composition are among the cellular and molecular structures that can be studied under an array of conditions. This approach not only enables but also improves and accelerates the identification of potential therapeutic targets. For example, mass spectrometry-based proteomics approaches applied to the study of *C. neoformans* have identified numerous proteins associated with the activation and production of virulence factors, key host-pathogen interactions, mechanisms of antifungal resistance, and biomarkers (147–153).

On the other hand, proteomics studies may significantly influence preclinical drug discovery by studying the differences in protein activity due to the interaction with bioactive molecules, which gives information about the drug activity in a proper study model. Chemoproteomic approaches, such as compound-centric chemical proteomics, activity-based protein profiling, thermal proteome profiling (TPP), limited proteolysis-coupled mass spectrometry, and proteome drug affinity-responsive target stability, are valuable for the study of protein-drug affinity, protein activity, protein-drug complex stability, and altered protein folding, respectively (154–158). For example, TPP enables the identification of on- and off-targets of a compound, which is an important task to avoid safety risks, as sometimes the drug may inhibit essential proteins for the host, causing toxic effects (158, 159). In this sense, the approach works based on giving a thermal shift to the sample with proteins in a complex with the drug that will display increased stability and resistance to thermal stress for identification by mass spectrometry. The application of mass spectrometry-based proteomics combined with drug discovery against fungal pathogens is an emerging field of study with promising potential to uncover novel antifungal therapeutics with increased specificity for the fungus and reduced side effects toward the host.

### Computational approaches to drug discovery

**Fig 2:**
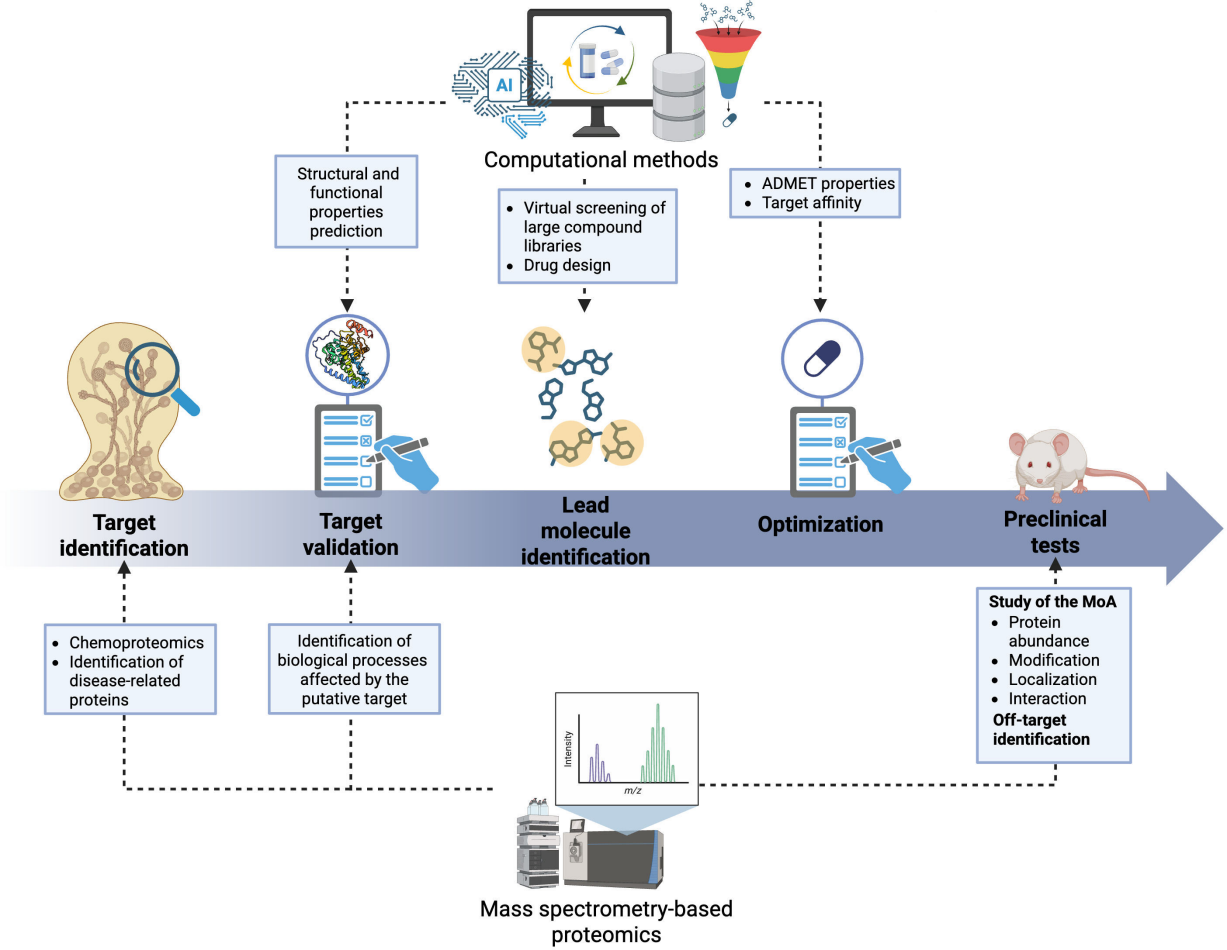
Mass spectrometry-based proteomics and computational methods applicable in the drug discovery process. MoA, mechanism of action.

Recent advances in computational power and algorithm development are driving new avenues in the drug discovery process (160–162). Techniques, such as computer-aided drug design (CADD), molecular docking, virtual HTS (vHTS), and AI enable faster target identification and validation, lead identification and optimization, and studies in the preclinical phase (163–165). For example, CADD has broad applications and usage after target identification to increase the hit rate for discovering new drugs and reduce the cost of research and development, which can cost billions when using traditional HTS of large compound libraries (166, 167). CADD can follow a structure- or ligand-based approach depending on information availability. For a structure-based approach, interaction energies can be calculated for all tested compounds, preferring low energy interactions. On the other hand, a ligand-based approach uses information from known bioactive and inactive molecules to screen for similarity or to construct molecules with predicted activity through quantitative structure-activity relationship models (168). CADD is often used prior to HTS as a vHTS strategy to reduce the number of molecules used in an experiment, supporting the focusing of time and resources on molecules with likely desired effects and properties (169). CADD may also be used to optimize ADMET properties after identifying a lead compound, *de novo* design of molecules based on combining functional groups in the molecular structure, or integration of known bioactive fragments into new chemotypes (170). Recently, computational innovation has developed novel AI models, such as AlphaFold, which are suitable for predicting protein structures and composing desired target structures (139). This computational approach has proven beneficial for performing *in silico* experiments within a shorter timeframe compared to traditional methods, such as X-ray crystallography, cryo-electron microscopy, and nuclear magnetic resonance (139). Moreover, AlphaFold 2 and AlphaFold 3 enable the prediction of protein-protein and protein-ligand interactions, respectively, allowing a plethora of uses within drug discovery (171, 172). These outputs can be combined with software platforms for molecular docking to predict how a small molecule will bind to a larger molecule by simulating possible orientations and conformational placements (173). Together, these computational-based approaches enhance the throughput and power of the drug discovery pipeline and propose innovative applications in combination with mass spectrometry-based proteomics (Fig. 2) .

## COMBINING PROTEOMICS AND COMPUTATIONAL APPROACHES TO CHARACTERIZE AND OVERCOME FUNGAL BIOFILMS

### Current applications and future strategies against *C. neoformans* biofilms

The presence of cryptococcal biofilms is related to severe cases of meningoencephalitis, colonizing both host tissue and medical devices for the brain (e.g., ventricular shunts), exerting a high resistance against common antifungal drugs used in the treatment of this disease, especially those of the azole class (174, 175). Early research into cryptococcal biofilms using mass spectrometry-based proteomics assessed the molecular processes for developing biofilms (176). The study compared proteomes of *C. neoformans* planktonic cells to cells within biofilms to identify 2,082 and 1,965 proteins, respectively. Differences in metabolic processes between the cell types were reported, suggesting a shift from the tricarboxylic acid cycle-related pathways to fermentation pathways for energy production in the fungal biofilms. Biofilm formation was further emphasized by the elevated abundance of enzymes, such as glutamate synthase, lactoylglutathione lyase, glutamate synthase, and 1-pyrroline-5-carboxylate dehydrogenase. Additionally, the study also determined that proteins involved in oxidative stress response and adhesion showed higher production levels within biofilms. These findings correlate with known biofilm characteristics of increased adhesion and resistance properties. The study also defined potential targets to impair biofilm formation by affecting these biological processes.

An expansion upon this study applied mass spectrometry-based proteomics to explore differences between cryptococcal species (i.e., *C. neoformans* and *Cryptococcus gattii*) in biofilm formation (177). Herein, the authors identified 1,819 proteins with 78% similarity between the strains to reveal a conserved strategy for biofilm formation. The cryptococcal biofilms support an adherent lifestyle by reducing the production of glycolytic proteins, such as glucose-6-phosphate isomerase and malate dehydrogenase. Conversely, the production of proteins associated with energy acquisition and reoxidation pathways, such as succinyl-CoA synthetase and cytochrome C oxidase, was elevated. Despite these similarities, important differences between the species were also noted, including an increased abundance of proteins involved in the electron transport chain, DNA binding, and transcription within *C. gattii*. Moreover, biofilms from *C. neoformans* showed higher levels of proteins associated with oxidoreductase activity, catabolic processes, and protein folding. Together, these studies use mass spectrometry-based proteomics to characterize cryptococcal biofilms and identify putative targets influencing drivers of biofilm formation but reveal a limited understanding of the complexities of cryptococcal biofilms. Future studies may focus on the identification of clinically relevant biofilm-associated proteins under diverse growth conditions and in the presence of host cells (e.g., macrophages) or from tissue samples collected in the clinic. Recent exploration into the interplay between the host and *C. neoformans* within brain tissue collected from a murine model of cryptococcal infection reveals new insights into modulators of fungal infection within immune cells and strategies to combat infection (178). This approach may also help prioritize putative druggable targets critical for biofilm formation within a medical setting. To evaluate these targets, future experiments could apply computational-based approaches to predict protein-ligand interactions with favorable binding affinities that would inhibit the target cryptococcal biofilm-associated proteins. The long-term goals of such experiments would lead to the prevention or disruption of cryptococcal biofilms from forming within patients and on medical devices.

### Current applications and future strategies against *A. fumigatus* biofilms

*A. fumigatus* biofilms are complex structures formed by packed filaments of cells embedded in a self-produced EPM. Such structures increase the difficulty of clearing infections and promote resistance to antifungals. Using quantitative proteomics, a study of the *A. fumigatus* biofilm formation under itraconazole pressure suggested potential molecular mechanisms driving azole resistance (179). In this study, the wild-type *A. fumigatus* strain, Af293, and an azole-resistant clinical strain, Shjt42b, were grown in media to promote biofilm formation. Additionally, the media contained different concentrations of itraconazole for each strain; thus, Af293 was grown at 0 and 0.25 µg/mL, while Shjt42b was grown at 0 and 256 µg/mL. As expected, changes in the production of enzymes involved in the ergosterol biosynthesis pathway were defined; specifically, Cyp51B (lanosterol demethylase) and Erg6 (sterol methyltransferase) showed higher abundance upon incubation with itraconazole. Oxidative stress response proteins, peroxiredoxin Aspf3 and catalase B, also showed elevated production in the biofilm during itraconazole treatment, suggesting a potential protection from the drug-induced oxidative stress. Together, the study highlighted putative targets to reverse azole resistance in *A. fumigatus* biofilms, with further characterization and validation needed.

Another study of the *A. fumigatus* biofilm proteome was performed through matrix-assisted laser desorption ionization-time-of-flight mass spectrometry for protein profiling of planktonic and sessile cells at different time points (24 and 48 h) (180). This approach enabled the identification of changes in protein abundance and, thus, correlated these changes to metabolic and molecular processes changing during the initial development of the biofilm structure and the further maturation stage. Similar to other studies, such as those for *C. albicans,* this work showed main differences in the metabolism of carbohydrates and amino acids. For instance, during the first 24 h, the sessile cells use carbohydrates as an energy source, whereas by 48 h (maturation), affiliated enzymes decrease in abundance, and formate catabolism-related proteins increase instead. This metabolic change has significant implications for chronic infections, also seen in other fungi (i.e., *C. albicans* and *C. neoformans*). Additionally, the production of bioactive molecules influences diverse *Aspergillus* spp. virulence determinants, such as the synthesis of secondary metabolites, which show higher abundance in mature biofilms. For example, proteins involved in gliotoxin synthesis, a small hydrophobic peptide that suppresses the innate and adaptive immune responses during infection, showed elevated production within mature biofilms.

Both studies collectively suggest significant cellular, molecular, and metabolic changes in *A. fumigatus* during biofilm formation, consistent with those observed in other fungi. Notably, these changes are associated with the biofilm phenotype and affect key biological processes, such as ergosterol synthesis, secondary metabolite synthesis, oxidative stress response, and DNA repair and replication. Understanding these changes is crucial as it can help explain biofilm resistance to the immune system and antifungal drugs. It also highlights potential targets for inhibiting biofilm formation and the associated virulence mechanisms, such as mycotoxins. Further studies could involve the use of targeted approaches to validate the key participation of such putative targets in biofilm formation, followed by elucidating the protein structure. This information can then be used to identify a ligand through computational approaches.

### Current applications and future strategies against *Candida* spp. biofilms

*Candida* biofilms are heterogeneous structures that embed yeast and hyphal cells to provide a protective layer against external stressors, such as the immune system and antifungal agents. Extensive studies using mass spectrometry-based proteomics toward *Candida* spp. biofilms have identified important protein-level drivers of biofilm composition and resistance, proposing new opportunities to target these structures and clear infections with improved efficacy (181, 182). Importantly, proteomics studies pertaining to *Candida* spp. biofilms are more prevalent than the other priority fungal pathogens (e.g., *C. neoformans* and *A. fumigatus*), providing an exciting opportunity to extend the approaches, techniques, and findings beyond *Candida* spp. to drive new studies in diverse fungi. For example, mass spectrometry-based proteomics of planktonic and sessile *C. albicans* cells after 24 h of incubation at 37°C detected 64 proteins with differential production during biofilm formation (183). The study highlighted changes in proteins associated with stress response, transcription, replication, RNA processing, proteolysis, transport, signal transduction, cell wall, plasma membrane, and metabolism. Notably, activation of transduction signals by cAMP-PKA and MAPK was linked to the morphological switching from yeast to hyphae, as well as the increased production of proteins involved in cell wall structure (e.g., Sun41, Pir1, and Csh1) and elevated glycosylation events. A similar study on the proteomes of *C. albicans* planktonic cells and biofilms detected 280 and 449 proteins, respectively (184). Biofilm-associated proteins demonstrated a higher abundance of proteins associated with the endomembrane system, such as Kar2, Mon2, and Pma1, as well as the mitochondrion and cytoplasm. A comparison of biological processes common and distinct between the biofilms and planktonic cells highlighted significantly more proteins associated with cellular homeostasis and vesicle-mediated transport connected to biofilm formation. For instance, Vps34 (phosphatidylinositol 3-kinase), an enzyme critical to *Candida* vacuolar protein transport and virulence, was identified exclusively within the biofilm proteome. Assessment of a genetic deletion strain of vps34 demonstrated a role for the protein in morphological switching from yeast to hyphae, essential for the infection process of *Candida* spp., suggesting Vps34 as a putative antibiofilm target. Differences in proteins related to lipid metabolism were also detected, with the biofilms displaying approximately 2.5-fold higher identifications than planktonic cells. Lipid metabolism is a key process for fungal membrane maintenance, as demonstrated by the inhibition of proteins involved in the ergosterol biosynthetic pathway, which is the mechanism of action for azoles and allylamines. Targeting enzymes and other proteins involved in these pathways during biofilm formation represents a potential therapeutic strategy for treating biofilms.

Further investigation into biofilm regulation used an isobaric tag for relative and absolute quantitation (iTRAQ)-based quantitative proteomics approach on *C. albicans* biofilms of haploid (GZY792 and GZY803) and diploid (SC5314 and BWP17) strains (185). Notably, the haploid strain, GZY803, is more sensitive to amphotericin B than the other strains, and differences in proteins involved in stress response and carbohydrate metabolism were defined within the haploid strain. Additionally, analysis of antioxidant proteins (involved in stress response) identified Ahp1, a cell wall peroxidase, with lower abundance in the haploid strain. For further validation, deletion of *ahp1* in BWP17 was performed, resulting in increased susceptibility to amphotericin B. Furthermore, induced overexpression of *ahp1* in the haploid strain increased resistance in the biofilm phenotype, demonstrating the critical role of Ahp1 in stress response induced by amphotericin B. This suggests that Ahp1 could be a potential drug target for combinatory therapies with this antifungal.

An activity-based proteomics approach was used to study virulence determinants (i.e., exoenzymes) in *Candida* spp. biofilms (186). In this study, the proteolytic activity of planktonic and sessile cells was characterized through multiplex substrate profiling by mass spectrometry. This involved exposing the fungal cultures (i.e., biofilms and planktonic cells) to a library of peptide substrates, and time-dependent peptide cleavage products were identified using tandem mass spectrometry. Notably, the biofilm cells showed a higher proteolytic activity and a distinct substrate specificity profile, suggesting the activity of specific proteases. Thereby, targeted proteomics was used to identify the putative proteases participating in the biofilm phenotype, with Sap5 (Candidapepsin-5) and Sap6 (Candidapepsin-6) identified as the biofilm-specific proteases with elevated abundance. Furthermore, deletion of *sap5* and *sap6* caused a reduction in biofilm formation, demonstrating their potential as therapeutic targets.

Another approach explored the relationship between *C. albicans* and other pathogens within a polymicrobial biofilm setting, such as the pathogenic bacteria *Streptococcus mutans*, which are linked to severe childhood caries (187). This study used iTRAQ-based proteomics and RNA-Seq to study and validate molecular mechanisms orchestrating the cross-kingdom interaction between *C. albicans* and *S. mutans* biofilms, which enhance persistent biofilm structures. The study showed a synergy in carbohydrate metabolism of both species, where *S. mutans* breaks down sucrose into glucose and fructose, which *C. albicans* can use to accelerate its growth rate and increase acid production. For *S. mutans*, the exoenzyme glucosyltransferase B (GtfB) has a prominent role in the attachment of bacterial cells to the fungal cell wall during biofilm formation. Further characterization of GtfB revealed a secondary role in the breakdown of sucrose, providing this metabolically available carbohydrate precursor. Beyond cross-domain polymicrobial biofilms, mixed *Candida* spp. biofilms may also occur and influence biofilm properties. A recent study used nano-tandem mass spectrometry to define proteome profiles of extracellular vesicles (EVs) secreted by *Candida* during biofilm formation and infection (188). A comparative proteomics study across *C. albicans*, *C. auris*, *Candida tropicalis*, *Candida parapsilosis*, and *Candida glabrata* showed 36 common cargo proteins in the EVs of all species. Further validation through gene deletion strains demonstrated that most of these cargo proteins were important for biofilm formation. This study highlights the potential of targeting EV-mediated processes in antifungal therapy, offering a new direction to develop a broad-spectrum antifungal against *Candida* biofilms.

Studies of *Candida* biofilm formation during exposure to common antifungals and bioactive compounds using quantitative proteomics also provide insights into biofilm regulation and modes of action. For instance, proteomic profiling of *C. albicans* during biofilm formation in the presence of caspofungin, an echinocandin-type antifungal drug used to treat invasive candidiasis, defined increased caspofungin resistance of biofilms compared to planktonic cells, surviving a concentration of 128 µg/mL (189). The proteomes of both phenotypes were compared with and without the drug, with each phenotype displaying distinct proteome profiles during caspofungin treatment. Notably, the biofilm showed twofold higher protein levels than the planktonic cells, highlighting the enhanced protection properties of biofilms. Specifically, proteins involved in cell wall integrity, stress response, and metabolic activity were elevated in biofilms treated with caspofungin. In another study exploring the proteome response to antifungals, the treatment of *Candida* spp. biofilms with zosteric acid, a secondary metabolite of the seagrass *Zostera marina*, was evaluated (190). This study showed a change in the production of 10 proteins involved in cell wall integrity and the adhesion of hyphae, which are important for biofilm attachment to the surface upon treatment. Using a similar approach, mass spectrometry-based proteomics unraveled the mechanism of action for the antibiofilm activity of myristic acid (synthesized by *Myristica fragrans*) during incubation with *Candida* biofilms (191). This suggests a reduction in proteins involved in distinct biological processes, such as oxidative stress, ergosterol synthesis, and sphingolipid metabolism, upon treatment. Moreover, mass spectrometry-based proteomics also investigated the mechanism of action of oleic acids against *C. albicans* biofilm formation to highlight dysregulation of morphology transition, stress response, and energy production of the biofilm (192).

Although rather limited to date, the promise and potential of computational approaches to advance knowledge of fungal biofilms and discover novel antifungal targets exist. For instance, recently, AlphaFold was used to predict protein structures involved in adhesion and invasion of *C. albicans*, crucial properties for biofilm formation (193). In this study, structures of stress response proteins, including Hog1, Tsa1, Trx1, Gpx3, Sod1, and Yhb1, were elucidated using AlphaFold. The predicted structural information was then used for a vHTS with the TIPdb database, employing domain analysis and molecular dynamics. The result was the identification of top-scoring ligands predicted to bind to the targeted proteins. The use of molecular dynamics in this study also supported the de-prioritization of low-stability therapeutic targets, paving the way for future studies to focus on more promising targets. This study highlights the use of computational approaches to accelerate the process of target structure elucidation and further drug screening, with potential applications to other putative targets identified and characterized in mass spectrometry-based proteomics studies.

## CONCLUSIONS AND FUTURE DIRECTIONS

Fungal biofilms are colonizing communities of microbial cells related to progressive and persistent infections, which are difficult to treat with the currently available array of antifungal drugs. Therefore, there is a necessity for the development of new antifungal drugs capable of inhibiting biofilm formation by the fungal pathogens discussed in this review. As defined by the WHO, *C. neoformans*, *A. fumigatus*, *C. albicans*, and *C. auris* are critical priority pathogens with implications for global health. By increasing our understanding of biological mechanisms regulating virulence factor production as well as antifungal resistance, both processes that benefit from biofilm formation, we aim to identify new anti-biofilm targets and propose novel antifungal drugs. To date, research into *Candida* spp. biofilms far surpasses that of other fungal pathogens and provides a roadmap for applications across pathogens, conditions, and diverse biological questions.

Mass spectrometry-based proteomics approaches have had a high impact through the identification of essential processes activated during biofilm formation as potential therapeutic targets (Table 1). From the studies performed to date and included within this review, heat shock protein 70 was elevated in abundance upon biofilm formation in *C. neoformans*, *A. fumigatus*, and *C. albicans*. Notably, additional cross-pathogen biofilm-associated proteins likely exist, but such identification may be limited by differences in culturing and sample processing and measurement techniques, as well as fewer mass spectrometry-based biofilm studies within *C. neoformans* and *A. fumigatus*. These proteins, primarily associated with metabolic shifts, oxidative stress response, adhesion, and, in some cases, morphological switches, serve as promising targets for further drug development. Moreover, mass spectrometry-based proteomics discoveries highlight potential targets for further drug development. Future steps include integrating computational tools, such as AlphaFold, with proteomics data to leverage identification of potential ligands by *de novo* drug design, vHTS, or traditional HTS with synthetic or natural compound libraries. Moreover, proteomics approaches may elucidate a drug’s mechanism of action and identify on- and off-target effects, which is critical to antifungal safety. On the other hand, multi-omics integration of genomics, transcriptomics, proteomics, and metabolomics has emerged as a powerful approach to comprehensively address the dynamic drug discovery pipeline and tackle current limitations. Overall, the novel array of available analytical and computational tools accelerates the process of antibiofilm drug discovery, paving the way for innovative applications and discoveries to combat biofilm-associated fungal infections.

**TABLE 1:** Proteins with elevated production during biofilm formation in *C. albicans*, *C. neoformans*, and *A. fumigatus*

Species	Biological process	Protein (UniProt ID)	Reference(s)
*C. albicans*	Cell wall	Csh1 (Q59QH2), Pir1 (Q59SF7), Sun41 (Q59NP5)	(183)
	Cell membrane	Cbp1 (P31225)	(183)
	Nucleotide metabolism	Rnr1 (Q5A0N3), nicotinamide-nucleotide adenylyltransferase (Q5AL24), Imh3 (O00086)	(183)
	Vitamin metabolism	Snz1 (Q5AIA6)	(183)
	Carbohydrate metabolism	Glx3 (Q5AF03), Gna1 (O93806), Pdc11 (P83779), Eno1 (P30575), Adh1 (Q07288), Adh2 (O94038)	(181–183)
	Electron transport chain	Atp14 (A0A1D8PHL7), Atp20 (Q59M30), Lfd6 (A0A1D8PD78)	(183)
	Tricarboxylic acid cycle	Mdh2 (P40926)	(181)
	Amino acid metabolism	Met15 (Q59US5), Met6 (P82610), Leu1 (A0A1D8PRP8), Ade17 (Q5A6R2), Aat1 (C6KTD0), Ilv2 (A0A1D8PJF9)	(181, 182)
	Lipid metabolism	Erg20 (A0A1D8PH78), Ole1 (Q5A747), Ino1 (P11986)	(181, 182, 184)
	Stress response	Crd2 (Q9P457), **Hsp70 (P41797**)	(181, 182)
	Transcription	Leo1 (G1UA63)	(183)
	RNA processing	Ago1 (A0A1D8PMK0)	(183)
	Translation	Tif4631 (A0A1D8PI73), Prt1 (Q5AGV4)	(183)
	Post-translational modification	Srp40 (A0A1D8PSB9), Wbp1 (A0A1D8PF22)	(183)
	Proteolysis	Rpn1 (A0A1D8PFL7)	(183)
	Transport	Hgt6 (Q5AD47)	(183)
	Mitochondrial	Mis11 (Q59SM8), Shm2 (O13426), Tuf1 (A5DN78)	(182)
	Miscellaneous	Hem13 (Q59MR4)	(182)
*C. neoformans*	Cell membrane	Mid2 domain-containing protein (J9VEM7), UBA/TS-N domain-containing protein (J9VQZ2)	(176)
	Nucleotide metabolism	Endoribonuclease L-PSP (J9VTD0), adenylyl-sulfate kinase (J9VMZ3), H/ACA ribonucleoprotein complex subunit CBF5 (J9VKI8)	(176)
	Carbohydrate metabolism	Glucose-6-phosphate 1-epimerase (J9VQU0), pyruvate dehydrogenase X component (J9VQH7), mannose-6-phosphate isomerase (J9VQA8)	(176)
	Electron transport chain	Adrenodoxin-type ferredoxin (J9VTR8), cytochrome c oxidase subunit 4 (J9W241), ubiquinone (J9W0I3)	(176)
	Amino acid metabolism	Glutathione reductase (J9VS68), PLP phosphatase (J9VRU6), transaminase (J9VQ60), lactoylglutathione lyase (T2BPJ4), uricase (J9VS03), TauD (J9VTG4), peptide-methionine (S)-S-oxide reductase (J9VXD1), glutamine synthetase (J9VJ14), glutamate dehydrogenase (J9VTK1), multifunctional fusion protein (J9W469)	(176)
	Lipid metabolism	Delta-9-desaturase (J9VVV4), dienelactone hydrolase domain-containing protein (J9W219), fatty acid synthase subunit beta (J9VVH4)	(176)
	Secondary metabolite biosynthesis	Metallo-beta-lactamase domain-containing protein (J9VT40), aldo-keto reductase (J9VRB4), aldose reductase (J9VWX4)	(176)
	Stress response	Sod1 (J9VLJ9), D-lactate dehydratase (J9W1G1), peroxiredoxin (J9VQF1), Cat (J9VJW0), **Hsp70 (J9VW24),** stress-induced-phosphoprotein 1 (J9VD75)	(176)
	Transcription	Zn (2)-C6 fungal-type domain-containing protein (J9VKK5)	(176)
	RNA processing	Uncharacterized (J9VGM4), small nuclear ribonucleoprotein G (J9VXE3), exoribonuclease phosphorolytic domain-containing protein (J9VKI9), nucleolin (J9VLN9), RRM domain-containing protein (J9VQ69)	(176)
	Translation	Large ribosomal subunit protein mL49 (J9VHU7), large subunit ribosomal protein L7 (J9W1A2), elongation factor 1-beta (J9VQK8), 40S ribosomal protein S12 (J9VID8), L14e (J9VSL1), polyadenylate-binding protein (J9VUR9), isoleucine–tRNA ligase (J9VDL3), tryptophan–tRNA ligase (J9VW18)	(176)
	Proteolysis	Methionine aminopeptidase (J9VJ52), extracellular metalloproteinase (J9VXZ9), peptide hydrolase (J9VWM8), leukotriene A-4 hydrolase/aminopeptidase (J9VNA2), dipeptidyl-peptidase V (J9VRU3),	(176)
	Transport	Ncs2 family nucleobase:cation symporter-2 (J9VJM2), GDP/GTP exchange factor Sec2 N-terminal domain-containing protein (J9VQD5)	(176)
	Cytoskeleton	Arp2/3 complex 20 kDa subunit (J9VL82), actin binding protein (J9VQ75), actin (P48465)	(176)
	Mitochondrial	Uncharacterized (J9VW88), Afg3 family protein (J9VGL8), cytochrome c oxidase assembly protein subunit 17 (J9W447), uncharacterized (J9VRB2), Ccp1 (Q6URB0)	(176)
	Transduction signaling	Calmodulin (J9VTH9)	(176)
	Miscellaneous	Cia1 (J9VZX3), fungal-type protein kinase domain-containing protein (J9VI35), universal stress protein (J9VQD7), RRM domain-containing protein (J9VG46), DUF6987 domain-containing protein (J9VK61), uncharacterized (J9VFP1), SnoaL-like domain-containing protein (J9W0Q8), BchC (J9VU15), cytoplasmic protein (J9VSP6), uncharacterized (J9VND1), cytoplasmic protein (J9VIF7), eisosome component PIL1-domain-containing protein (J9VQC9), oxidoreductase (J9VR10)	(176)
*A. fumigatus*	Lipid metabolism	Cyp51A (Q4WNT5), Cyp51B (E9QY26), Erg6 (Q4W9V1), Plb1 (P0C957), Plb3 (P0C958)	(179)^*a*^
	Amino acid metabolism	Methionine synthase (Q4WNY2)	(180)
	Nucleotide metabolism	Adk1 (Q4WJ21)	(179)^*a*^
	Secondary metabolite biosynthesis	GliT (E9RAH5), FtmOx1 (Q4WAW9)	(179, 180)^*a*^
	Stress response	Aspf3 (O43099), CatB (Q92405), Hsp90 (P40292), Tel1 (Q4WVM7), Ssc70 (Q4X1H5), **Hsp70 (Q4WCM2**)	(179, 180)^*a*^
	Translation	Tif6 (Q4WTT3)	(179)^*a*^
	Proteolysis	Dipeptidyl-peptidase 5 (P0C959)	(180)
	Transport	Mdr1 (Q4WTT9), AbcA (Q4X006), AbcC (E9RBG1)	(179)^*a*^
	Mitochondrial	Ccp1 (Q4WPF8)	(179)^*a*^

^*a*^ The study used comparative proteomics between biofilms treated with different concentrations of itraconazole. Proteins shared among the three fungal pathogens are in bold.
